# Editorial

**Published:** 1867-04

**Authors:** 


### Editorial.

CONVENTION OF DELEGATES FROM MED. COLLEGES.—As indicating the importance we attach to the movement for such a convention, we copy, in place of new editorial matter, the following report of our remarks, originally furnished to the *Med. and Surg. Reporter*, of Philadelphia:—

During the past year the subject of Medical College Organization and Instruction has been discussed with more freedom than usual in some of the leading medical journals of our country, as well as at the last annual meeting of the "American Medical Association." It will be remembered by our readers that, at the meeting just named, a proposition was made to endorse the action of a convention previously held in Cincinnati, composed of delegates from several medical colleges in the West and North-west, which had recommended a more uniform rate of lecture fees, and the extension of the annual lecture term to six months. The discussion that followed this proposition was participated in by Drs. D. H. Storer, of Boston; M. B. Wright, of Cincinnati; Worthington Hooker, of New Haven; N. S. Davis, of Chicago; and others.

It was claimed by the last named gentleman that the evils connected with our present system of medical education could not be remedied by simply lengthening the annual lecture terms and adopting any given rate of lecture fees.

However proper these measures might be, their influence would not reach the real source of most of the evils of which the Profession complain—namely, the graduation and admission into the ranks of the Profession of young men whose general education, mental discipline, and medical attainments are all extremely defective. These result, not from the rate of lecture fees, or the length of the lecture terms in our medical schools, but from the three following sources: First, the entire absence of all provision for securing any standard of preliminary education before entering upon the study of medicine. Second, the equally entire absence of all provision for securing a proper method and order of progress in the study of medicine itself. And third, the shortness of the lecture terms, and the insufficient means for clinical instruction in many of the schools. He represented the two first as *radical faults*, entering into the very foundation of our present system of medical college instruction, and vitiating all its practical results. They were violations of the most important principles, universally recognized in all other departments of education, in this and in all other civilized countries.

If a young man applies for admission to any literary college, or even high school, he is straightway subjected to an examination, sufficient to test his knowledge of the elementary branches of learning. And why? Simply because it is assumed, on the plainest principles of common sense, that a knowledge of the elementary branches is essential to a proper understanding of the higher and more intricate. It is assumed that a knowledge of arithmetic, grammer, geography, etc., must prepare the way for the higher mathematics, philosophy, history, rhetoric, etc.; and that a certain degree of mental discipline and habits of study are necessary to enable the student to make adequate advancement in his collegiate course.

In our present system of medical education all this is ignored, and whoever presents himself with the required fee is admitted to our medical colleges, without an inquiry as to whether his mind has been disciplined by an hour of previous study, or whether he is capable of constructing two sentences grammatically, or not.

The absence of any provision for securing a proper method and progressive order of study after entering the medical colleges, developes evils equally palpable and mischievous. It bewilders the student by crowding daily upon his mind too great a variety of subjects, and by presenting them in a perfectly heterogeneous order; while it strongly encourages superficiality of attainments, and constant efforts to comprehend the practical departments to the neglect of the more elementary. If the faculty of any of our literary colleges should throw their freshmen, sophomore, junior, and senior classes all into one grand class, and undertake to drill them one hour on some study belonging to the freshmen year, the next hour on one belonging to the senior year, the third hour on one belonging to the sophomore year, and so on, all the world would regard them as better fitted for a lunatic asylum than for teachers of youth. And yet, this would be exactly parallel to what is done, with one exception, in all the medical colleges in our country. The student just commencing his professional study, and not yet familiar with the naked bones of the skeleton, is placed side by side with the one who has studied two, three, or four years, and both listening during each day to lectures on histology, the elements of chemistry, physiology, and materia medica, interspersed with those on the details of practical medicine, surgery, and obstetrics.

The attempt to include an adequate course of instruction in the various branches of medical science, in five or six lectures per day, for sixteen or eighteen weeks, is not much less objectionable than the heterogeneous order in which the instruction is given.

He stated that nothing short of a full and careful revision of the whole system of Medical College education, could remove the foregoing defects. Such revision should be founded upon the following fundamental propositions:—

1st. A fair degree of general education and mental discipline is necessary to enable any young man to study medicine understandingly.

2d. A knowledge of the elementary branches of medicine, such as Anatomy, Physiology, etc., is essential as a preparatory step to the profitable study of Practical Medicine, Surgery, etc. Hence, a successive order of study is as necessary in the study of medicine as in general science.

3rd. The number of Professorships, and the length of the annual term in each college, should be such as to ensure a complete discussion or review of all the legitimate branches of medical science.

To place the system of medical college instruction in our country upon these self-evident propositions would require the universal adoption and enforcement of a reasonable standard of preliminary education for the medical student; the division of the whole subject of medical science into about twelve branches, *four* of which should be assigned in proper order to each of the three years required for medical study; the requirement of three courses of lectures instead of two, with a thorough examination of the student, at the close of each course, on those branches belonging to each year successively, and an increase of professorships in each college, corresponding with the whole number of branches to be taught, with an annual lecture term of not less than five full calendar months. To perfect and carry into practical operation a revision of our system of medical education, so important to the honor and usefulness of the profession, requires a free interchange of views and an honest concert of action among those connected with the medical colleges throughout the whole country. It cannot be accomplished by resolving here in this association that the college fees ought to be uniform, or that the annual lecture terms ought to be five, six, or seven months.

The speaker reminded the members of the association that almost every volume of their transactions, for the last sixteen years, contained resolutions of the same import as those now under discussion. If anything of practical value was accomplished it would have to be done by the college faculties themselves. He, therefore, moved, as a substitute for the resolutions under discussion, the following resolution:

"*Resolved*, That this Association earnestly requests the Medical Colleges of the country, to hold a convention for the purpose of thoroughly revising the present system of Medical College instruction, and that a committee be appointed to aid in carrying this resolution into effect."

---

Public Commencement of Chicago Medical College.—The commencement exercises of this institution were held on the 4th and 5th of March. The afternoon of the first day was occupied by reading Theses by some of the candidates for graduation, and the public examination of all the candidates in class. At 3 o'clock P. M., of the second day, the college hall was well filled with an appreciative audience. The exercises were opened with prayer by the Rev. Dr. R. M. Hatfield, of Chicago. The President of the Faculty, Prof. N. S. Davis, after stating briefly the peculiarities in the organization of the college, and the important educational advantages they were designed to afford, had the Secretary call the list of junior students who had undergone examinations in those branches of medical science embraced in the junior course, and delivered to each of them a certificate indicating their progress, accompanied by a few words of earnest exhortation, continued diligence and fidelity in the further prosecution of their professional studies.

The Secretary then called the list of candidates for graduation as follows:

E. F. Baker, John W. Barlow, Thomas S. Bond, Charles E. Crocker, John T. Curtiss, M. T. Didlake, Peter Epler, John G.

Fredighe, David J. Hussey, J. W. Hutchinson, Isaac R. Lane, Elmer T. Lawrence, Wm. Martin, Theron Nichols, Henry P. Oggel, Thomas D. Palmer, Wesley Park, J. A. Parmenter, D. Patton, F. A. Richards, Chester Reeder, R. R. Resseque, David Robertston, W. L. Secumb, D. A. Sheffield, E. T. Twining, M. L. Whitman, John Whelan, and the President conferred upon each the degree of Doctor of Medicine, accompanied by an impressive charge in relation to the privileges conferred upon them, and the duties and responsibilities they had assumed.

It was then announced that the Honorary Degree of Doctor of Medicine had been conferred upon Drs. D. W. Beebee, of Elizabeth, Ill.; Asher Goslin, of Olney, Ill.; Wm. Law, of Shullsburg, Wis.; N. Holton, of Buda, Ill., and Joseph P. Johnston, of Lynnville, Ill.

It was also announced that the Committee of the Faculty, to whom had been referred the Inaugural Theses, had awarded the premium for the best Thesis to John G. Fredighe, of Chicago, and for the second best to Theron Nichols, of Wisconsin.

R. S. Patterson, M.D., Professor of Medical Jurisprudence, was then introduced to the audience, and delivered an appropriate and highly interesting valedictory address.

Notice was given that the Summer Reading and Clinical Term in the College would commence on the second Monday in March and continue until the first Monday in July.

The exercises were closed with a benediction by Rev. Dr. Hatfield.

In the evening the graduates, students, and alumni of the College, the Faculty, and members of the Chicago Medical Society, enjoyed a very pleasant social entertainment at the residence of the President of the College.

---

Alumni Association.—Under the head of Proceedings of Medical Societies, in this number of the Examiner, will be found an account of the formation of the "Alumni Association of Chicago Medical College," with its Constitution and By-Laws. We invite to this the special attention of all the graduates of the college since its organization in 1859, and hope all who are now living will promptly respond to the invitation to become members of the Association.

Such an organization not merely tends to perpetuate friendship, social intercourse, and unity of feeling among the alumni of a common Alma Mater, but it is equally efficient in keeping alive a generous emulation in the pursuit of knowledge, in the attainment of professional eminence, and the maintenance of professional honor. And we can assure the alumni who may unite with the Association, that their Alma Mater will open wide its doors to welcome them at each returning anniversary meeting.

---

### AMERICAN MEDICAL ASSOCIATION.

---

OFFICE OF PERMANENT-SECRETARY,
WM. B. ATKINSON, M.D.,
*215 Spruce St., Philada.*

The Eighteenth Annual Meeting of the American Medical Association will be held in Cincinnati, on Tuesday, May 7th, 1867, at 11 o'clock A.M.

The following Committees are expected to report:—

On Quarantine, Dr. Wilson Jewell, Pa., Chairman.

On Ligature of Subclavian Artery, Dr. Williard Parker, N.Y., Chairman.

On Progress of Medical Science, Dr. Jerome C. Smith, N.Y., Chairman.

On the Comparative Value of Life in City and Country, Dr. Edward Jarvis, Mass., Chairman.

On Drainage and Sewerage of Cities, &c., Dr. Wilson Jewell, Pa., Chairman.

On the Use of Plaster of Paris in Surgery, Dr. Jas. L. Little, N.Y., Chairman.

On Prize Essays, Dr. F. Donaldson, Md., Chairman.

On Medical Education, Dr. S. D. Gross, Pa., Chairman.

On Medical Literature, Dr. A. C. Post, N.Y., Chairman.

On Instruction in Medical Colleges, Dr. Nathan S. Davis, Ill., Chairman.

On Rank of Medical Men in the Army, Dr. D. H. Storer, Mass., Chairman.

On Rank of Medical Men in the Navy, Dr. W. M. Wood, U.S.N., Chairman.

On Insanity, Dr. Isaac Ray, R.I., Chairman.

On American Medical Necrology, Dr. C. C. Cox, Md., Chairman.

On the Causes of Epidemics, Dr. Thomas Antisell, D.C., Chairman.

On Compulsory Vaccination, Dr. A. N. Bell, N.Y., Chairman.

On Leakage of Gas-Pipes, Dr. J. C. Draper, N.Y., Chairman.

On Alcohol and its Relations to Man, Dr. J. R. W. Dunbar, Md., Chairman.

On the Various Surgical Operations for the Relief of Defective Vision, Dr. M. A. Pallen, Mo., Chairman.

On Local Anæsthesia, Dr. E. Krackowitzer, N.Y., Chairman.

On the Influence upon Vision of the Abnormal Conditions of the Muscular Apparatus of the Eye, Dr. H. D. Noyes, N.Y., Chairman.

On the Comparative Merits of the Different Operations for the Extraction of Vesical Calculi, Dr. B. J. Raphael, N.Y., Chairman.

On the Therapeutics of Inhalation, Dr. J. Solis Cohen, Pa., Chairman.

On the Deleterious Articles used in Dentistry, Dr. Augustus Mason, Mass., Chairman.

On Medical Ethics, Dr. Worthington Hooker, Con., Chairman.

On the Climatology and Epidemics of Maine, Dr. J. C. Weston.
of New Hampshire, Dr. P. A. Stockpole.
Vermont, Dr. Henry Janes.
Massachusetts, Dr. Alfred C. Garratt.
Rhode Island, Dr. C. W. Parsons.
Connecticut, Dr. B. H. Catlin.
New York, Dr. E. M. Chapman.
New Jersey, Dr. Ezra M. Hunt.
Pennsylvania, Dr. D. F. Condie.
Delaware, Dr. — Wood.
Maryland, Dr. O. S. Mahon.
Georgia, Dr. Juriah Harriss.
Missouri, Dr. Geo. Engelman.
Alabama, Dr. R. Miller.
Texas, Dr. Greensville Dowell.
Illinois, Dr. R. C. Hamil.

Indiana, Dr. J. F. Hibberd.
District of Columbia, Dr. T. Antisell.
Iowa, Dr. J. W. H. Baker.
Michigan, Dr. Abm. Sager.
Ohio, Dr. J. W. Russell.

Secretaries of all medical organizations are requested to forward lists of their Delegates as soon as elected, to the Permanent-Secretary,

W. B. ATKINSON.

---

MORTALITY REPORT FOR THE MONTH OF FEBRUARY.—The following is the mortuary report of Health Officer Bridges, of this City, for the month ending March 1. The total number of deaths reported is 255, which is a reduction of 44 from the number of deaths last month:—

CAUSES OF DEATH.

| | | | |
|---|---|---|---|
| Accidents, | 6 | Fever, Congestive, | 3 |
| Asthma, | 1 | Fever, Remittent, | 2 |
| Abscess, | 2 | Fever, Scarlet, | 14 |
| Bronchitis, | 1 | Fever, Typhoid, | 10 |
| Cancer, | 3 | Hydrocephalus, | 4 |
| Cold, | 1 | Inflammation of Bowels, | 4 |
| Croup, | 8 | Inflammation of Brain, | 5 |
| Consumption, | 42 | Inflammation of Lungs, | 19 |
| Convulsions, | 18 | Inflammation of Stomach, | 1 |
| Childbed, | 5 | Inflammation not stated, | 1 |
| Congestion of Brain, | 7 | Killed, | 1 |
| Congestion of Lungs, | 2 | Marasmus, | 2 |
| Delirium Tremens, | 1 | Neuralgia, | 1 |
| Decline, | 2 | Old Age, | 6 |
| Diarrhœa, | 1 | Paralysis, | 1 |
| Diphtheria, | 6 | Pneumonia, | 1 |
| Dropsy, | 9 | Phthisis Pulmonalis, | 2 |
| Dysentery | 1 | Rheumatism, | 1 |
| Disease of Brain, | 1 | Stillborn, | 22 |
| Disease of Bowels, | 2 | Small-Pox, | 5 |
| Disease of Heart, | 4 | Teething, | 9 |
| Disease of Liver, | 2 | Whooping-Cough, | 1 |
| Disease of Lungs, | 2 | Worms, | 1 |
| Disease of Throat, | 1 | Gun-shot Wound, | 1 |
| Epilepsy, | 1 | Ulcer, | 1 |
| Erysipelas, | 2 | Unknown, | 6 |
| Total, | | | 255 |

AGES OF THE DECEASED.—Under 5 years, 118; over 5 and under 10 years, 17; over 10 and under 20, 10; over 20 and under 30, 35; over 30 and under 40, 31; over 40 and under 50, 16; over 50 and under 60, 8; over 60 and under 70, 10; over 70 and under 80, 6; over 80 and under 90, 2; unknown, 2. Total, 255.

NATIVITIES.

| | | | | | |
|---|---|---|---|---|---|
| Chicago, | 124 | Germany, | 30 | Scotland, | 2 |
| Other States, | 39 | Ireland, | 33 | Wales, | 1 |
| Canada, | 1 | Norway, | 3 | Unknown, | 5 |
| England, | 12 | Nova Scotia, | 1 | | |
| France, | 1 | Sweden, | 3 | Total, | 255 |

DIVISIONS OF THE CITY.

North,......... 67 | South,......... 81 | West,.........106 | Total,......... 255

MONEY RECIPTS FOR EXAMINER, FROM FEB. 21ST TO MAR. 22ND.—Drs. J. H. Judson $3, G. P. Martin 3, Brown H. Kimber 3, J. T. Curtiss 3, R. B. Treat 6, D. F. Morrow 6, H. H. Deming 3, Geo. R. Bibb 3, Geo. H. Scott 3, R. S. Addison 6, L. C. White 3, Wm. H. Sommers 3, M. Lindly 6, B. F. Brown 3, J. S. Sherman 3, Asahel Clark 3, S. L. Fuller 6, Jesse H. Foster 17, W. W. Duncan 4, James M. Hutchinson 3, A. A. Dunn 3.25, J. M. Larrabee 3, J. H. Reynolds 3, L. D. Tompkins 10, David B. Trimble 3, Jas. Hayton 3, R. J. Patterson 3, Andrew J. Miller 3, J. W. Lawrence 3, E. M. Blackburn 3.

